# U wave: an Important Noninvasive Electrocardiographic Diagnostic Marker

**Published:** 2005-01-01

**Authors:** Girish M P, Mohit Dayal Gupta, Saibal Mukhopadhyay, Jamal Yusuf, Sunil Roy T N, Vijay Trehan

**Affiliations:** Department Of Cardiology, G B Pant Hospital, New Delhi-110002, India

**Keywords:** Electrocardiogram, U wave

## Abstract

Study of U waves exemplifies important clinical role of noninvasive electrocardiography in modern cardiology. Present article highlights significance of U waves with a clinical case and also summarizes in brief the history of the same

Study of U waves has been more of academic importance rather than clinical application. Negative U waves when present may be of immense clinical importance. We describe here one such case where U waves were not only an early noninvasive marker for acute ischemia but they also disappeared after successful revascularization.

The U wave, named by Einthoven in 1903, is still a subject of debate with respect to its origin and clinical importance [1]. Three hypotheses addressing the genesis of U wave that have been put forth include late repolarization of Purkinje fibers, late repolarization of some other portions of left ventricle, and alteration in the normal action potential shape by after-potentials [2]. U waves have same polarity as T waves in normal subjects. Any alteration in the same with respect to T wave is of importance. A 50 year old male, chronic smoker, non hypertensive and non diabetic presented with unstable angina (Braunwald class IIIb2).Electrocardiogram showed normal sinus rhythm with upright T waves and no ST-T changes, but prominent inverted U waves in mid precordial leads (Figure 1a). Echocardiography showed normal LV systolic and diastolic function. Coronary angiogram revealed a discrete tight stenosis (90%) in the mid left anterior descending artery (Figure 2a). Coronary angioplasty with stenting was successfully carried out (Figure 2b). Immediate post procedure ECG revealed remarkable disappearance of U waves (Figure 1b).

Negative U waves have low sensitivity but high specificity for heart disease and they are recorded in approximately 1% of all electrocardiograms in general hospital [3]. Negative U waves at rest, may be the earliest marker of unstable angina and evolving myocardial infarction [3].  They may also be seen at rest in cases of hypertension, variant angina, congenital long QT syndromes, left ventricular enlargement, left anterior descending coronary artery disease and valvular heart disease (aortic and mitral valve disease) [3,4]. Others however showed that negative U waves during acute anterior wall myocardial infarction are useful in identifying patients with smaller infarction and better collateral circulation but they had no predictive value in localizing the diseased artery [5]. U waves on exercise may be seen in cases of significant left circumflex or right coronary artery disease with abundant collaterals [6].

The present case exemplifies the significance of negative U waves as the important sign of acute ischemia. We believe that clinical utility of U waves remains underutilized and more clinical studies are needed to exactly define their significance in the spectrum of coronary artery disease.

## Figures and Tables

**Figure 1:**
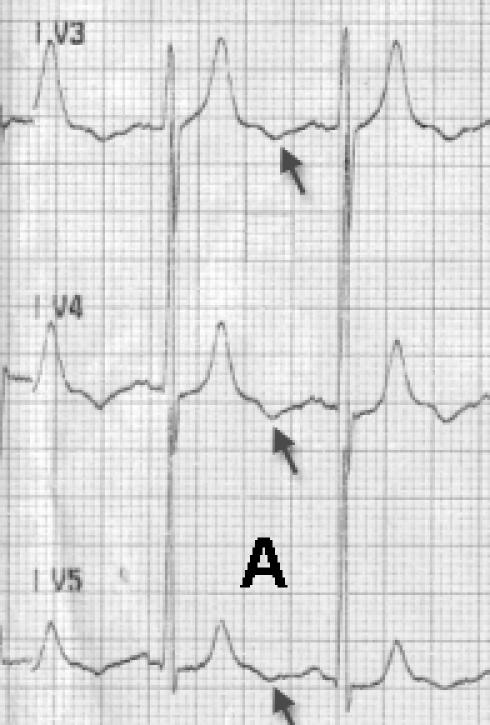

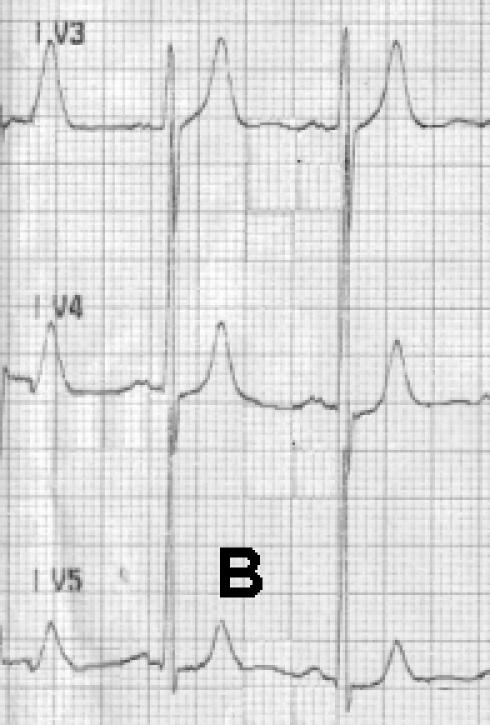
**A** Electrocardiogram showing prominent ‘U’ waves (arrows) in leads V3, V4, V5 in patient prior to intervention. **B** Post revascularization electrocardiogram showing dramatic disappearance of U waves with similar other findings

**Figure 2:**
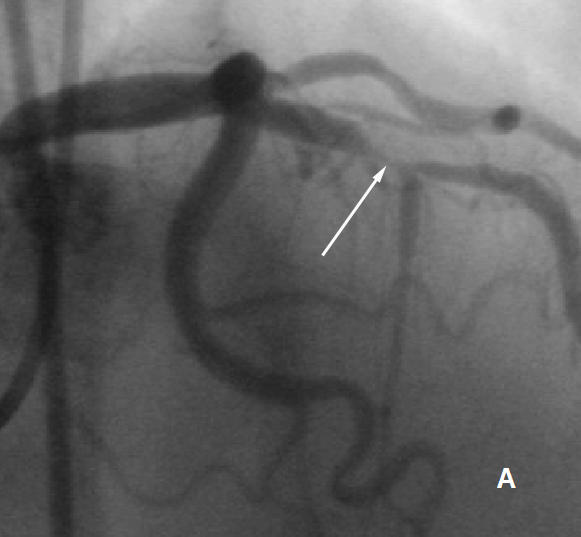

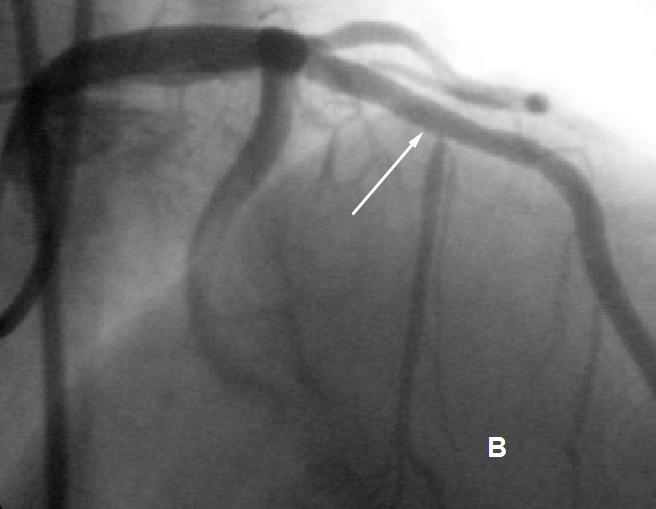
**A** Angiogram in Right anterior oblique cranial view showing tight stenosis in left anterior descending artery **B**  Post revascularization angiogram in Right anterior oblique cranial view showing TIMI 3 flow in left anterior descending artery

## References

[R1] Einthoven W (1903). Die galvanometrische registrierung des menschlichen Electrokardiogram. Pfluger’s Arch.

[R2] Bernardo DD, Murray A (2002). Origin on the electrocardiogram of U-waves and abnormal U-wave inversion. Cardiovasc Res.

[R3] Fisch C (1997). The clinical electrocardiogram: sensitivity and specificity. ACC Curr J Rev.

[R4] Gerson MC, McHenry PL (1980). Resting U-wave inversion as a marker of stenosis of the left anterior descending coronary artery. Am J Med.

[R5] Tamura A, Watanabe T, Nagase K (1997). Significance of negative U waves in the precordial leads during anterior wall myocardial infarction. Am J Cardiol.

[R6] Miwa K, Nakagawa K, Hirai T (2000). Exercise-induced U-wave alterations as a marker of well-developed and well-functioning collateral vessels in patients with effort angina. J Am Coll Cardiol.

